# Validation of the Turkish Version of the Professional Fulfillment Index

**DOI:** 10.5811/westjem.21199

**Published:** 2024-09-25

**Authors:** Merve Eksioglu, Ayca Koca, Burcu Azapoglu Kaymak, Tuba Cimilli Ozturk, Atilla Halil Elhan

**Affiliations:** * University of Health Sciences Fatih Sultan Mehmet Education and Research Hospital, Department of Emergency Medicine, Istanbul, Turkey; † Ankara University Faculty of Medicine, Department of Emergency Medicine, Ankara, Turkey; ‡ Ankara University Faculty of Medicine, Department of Biostatistics, Ankara, Turkey

## Abstract

**Introduction:**

Clinician burnout represents a significant occupational hazard among physicians, with a notably high prevalence among emergency physicians. The Stanford Professional Fulfillment Index (PFI) was developed to comprehensively assess various aspects of doctors’ work experiences, including professional fulfillment. In this study we aimed to validate the Turkish version of the PFI (T-PFI), a 16-item instrument designed to measure physicians’ professional fulfillment and burnout.

**Methods:**

In this cross-sectional study, we validated the T-PFI in two phases. The initial phase involved translating and culturally adapting the original PFI into Turkish. We evaluated the content validity of the translated version using item and scale content validity indices (I-CVI and S-CVI, respectively). The validated T-PFI was then distributed among a broad cohort of emergency physicians via an online survey to further assess its reliability and validity. The assessment tools included Cronbach α, confirmatory factor analysis, and content validity indices.

**Results:**

Of 1,434 physicians who were sent the survey, 425 fully completed it (29.6%). There was an almost equal distribution of 215 females and 210 males. Only 9.6% of the participants reported high levels of professional fulfillment, whereas a significant majority (79.1%) were susceptible to burnout. The Cronbach α values for the professional fulfillment and overall burnout scales were 0.87 and 0.90, respectively. The content validity was confirmed by I-CVI values exceeding 0.80 and an S-CVI/average relevance of 0.92. The confirmatory factor analysis demonstrated an acceptable model fit after adjustments.

**Conclusion:**

The T-PFI is a reliable and valid tool for assessing professional fulfillment and burnout among emergency physicians in Turkey. Effective interventions to mitigate burnout are essential to improve physician well-being in Turkish healthcare settings.

Population Health Research Capsule What do we already know about this issue?

*Emergency physicians experience high rates of burnout, negatively impacting their well-being and patient care.*

What was the research question?

*Is the Turkish version of the Professional Fulfillment Index (T-PFI) valid and reliable?*

What was the major finding of the study?

*The T-PFI showed strong internal consistency (Cronbach α: 0.87 for PF, 0.90 for burnout).*

How does this improve population health?

*The T-PFI offers a reliable measure of professional fulfillment and burnout, enabling healthcare organizations to monitor physician well-being.*

## INTRODUCTION

Burnout is a syndrome resulting from chronic workplace stress and overload.^1^ It is characterized by three dimensions: emotional exhaustion; depersonalization; and reduced personal accomplishment.^2^ Studies have shown that physicians experience higher rates of burnout and lower satisfaction with work-life integration compared to the general population.^3^ Emergency physicians, in particular, are highly susceptible to burnout, with rates surpassing those seen in other medical specialties.^3^ Research on the work experiences of emergency physicians has largely focused on individual and workplace factors contributing to burnout.^4^ ^,^ ^5^ Burnout not only affects clinicians’ health but also negatively impacts patient care outcomes and the overall functioning of healthcare systems.^6^ ^,^ ^7^

To effectively measure burnout among healthcare professionals, it is crucial to assess not only the negative aspects, such as emotional exhaustion and depersonalization, but also the positive aspects such as professional satisfaction, well-being, and occupational fulfillment.^8^ ^–^ ^11^ Unlike traditional burnout scales, the Stanford Professional Fulfillment Index (PFI) was developed to provide a comprehensive assessment of physicians’ work experiences.^10^ The PFI is a concise tool specifically designed for physicians, offering valuable insights into their well-being, quality of life, and productivity. It helps healthcare organizations evaluate both burnout and professional satisfaction levels, enabling the development of programs to enhance job satisfaction and overall well-being. However, for the PFI to be effective across different cultural contexts, it must be translated and adapted to ensure its validity and relevance. The PFI has been successfully adapted in countries such as Brazil and Japan, but further validation is needed to confirm its psychometric strength and cultural applicability.^12^ ^,^ ^13^

In this study we aimed to develop and validate the Turkish PFI (T-PFI) to assess the professional fulfillment and burnout levels of emergency physicians in Turkey. The T-PFI is based on the Stanford PFI and has been translated and adapted to fit the specific cultural and healthcare context of Turkey. We then evaluated the psychometric properties of the T-PFI to ensure its reliability and validity. Our goal was to provide a robust tool for accurately assessing and monitoring the professional well-being of emergency physicians in Turkey.

## METHODS

### Participants

The research team collaborated with the heads of high-volume emergency departments (ED) in prominent healthcare institutions across Turkey to recruit participants. Recognizing the potential for a low response rate due to the demanding schedules of emergency physicians, we employed several strategies recommended by the literature to improve response rates and mitigate non-response bias.^14^ These strategies included sending multiple reminders and using various communication platforms to distribute the survey link.

We invited emergency physicians affiliated with these organizations through emails, social media groups, and internal communication platforms, such as online messaging systems. To ensure clarity for potential participants, we provided a detailed explanation of the study’s purpose and objectives. Eligible participants included physicians actively working in EDs. While our primary aim was to identify burnout among these healthcare professionals, we expanded the study to include residents, attending physicians, consultant physicians, and faculty members. With this broader scope we aimed to capture a diverse range of perspectives, ensuring comprehensive representation of healthcare professionals within emergency medicine (EM).

We sent the survey to 1,434 medical doctors. Participants provided electronic informed consent before completing the survey, confirming their voluntary participation and understanding of the study’s objectives and procedures. To protect their privacy and encourage candid responses, we designed the survey to be anonymous. We included only fully completed questionnaires in the statistical analysis, resulting in a response rate of 29.6%.

### Procedure

We conducted this cross-sectional study in two phases to validate the T-PFI. In the first phase, we translated and culturally adapted the original PFI into Turkish, adhering to World Health Organization guidelines for linguistic accuracy and cultural relevance.^15^ Two bilingual emergency physicians performed the forward translation of the PFI from English to Turkish. A panel of experts, including three faculty members (one professor and two associate professors) and two emergency physicians with a special interest in physician well-being, reviewed and revised the translation to improve its cultural relevance.

To assess the content validity of the translated PFI, we calculated item and scale content validity indices (I-CVI and S-CVI). Ten independent experts rated item relevance on a four-point scale, with 1 or 2 indicating no relevance and 3 or 4 indicating relevance. We set acceptable thresholds at I-CVI >0.78 and S-CVI >0.90, based on guidelines for expert panels of 6–10 members.^16^

In the pre-test phase, we obtained feedback from five male and five female emergency physicians working in crowded EDs. Based on this feedback, we refined the instrument, resulting in the final version of the T-PFI (Appendix A). We then distributed the validated Turkish version to a broader cohort of respondents in EDs for further validation. The Institutional Review Board of the University of Health Sciences Fatih Sultan Mehmet Education and Research Hospital approved all procedures.

### Measures

The PFI, developed by Trockel et al, assesses professional fulfillment and burnout among physicians.^10^ The original PFI comprises 16 items categorized into two scales: the Professional Fulfillment (PF) scale (six items) and the Overall Burnout scale (10 items), measuring three dimensions: professional fulfillment; professional exhaustion, and interpersonal disengagement. Each item is evaluated using a five-point Likert scale (0 to 4). Higher scores on the PF scale (7.5 or greater) indicate higher professional fulfillment, while scores exceeding 3.325 on the burnout scale suggest potential burnout. The PFI, validated in 2018, evaluates emotional exhaustion, interpersonal disengagement, and professional achievement, expanding beyond traditional burnout dimensions to include intrinsic work components such as happiness, meaning, self-esteem, and satisfaction.^10^

### Data Analysis

We computed descriptive statistics for demographic characteristics and T-PFI scores. Mean scores for each item and dimension were calculated according to Trockel’s instructions.^10^ We evaluated the reliability of the T-PFI through internal consistency, which estimates the extent to which the constituent items of the scale are interrelated. The Cronbach α coefficient was used to assess internal consistency, with values above 0.70 indicating acceptability.^17^ ^,^ ^18^ We evaluated construct validity through confirmatory factor analysis (CFA) of responses to all 16 items, aiming to assess model fit. Factor loadings were calculated using maximum likelihood estimation. To evaluate the model’s goodness of fit, we considered several indices, including the chi-square statistic (χ^2^), root mean square error of approximation (RMSEA), comparative fit index (CFI), and the Tucker-Lewis index (TLI). The thresholds for these indices were as follows: TLI >0.90 (acceptable), >0.95 (excellent); CFI >0.90 (acceptable), >0.95 (excellent); RMSEA <0.08 (acceptable), <0.05 (excellent); and chi-square statistic divided by the degree of freedom (<3 acceptable).^19^ ^,^ ^20^ Error items were only correlated if they belonged to the same construct.

For validation studies, it is recommended to have a minimum of 50 respondents for each criterion and construct validation studies that involve calculating correlation coefficients. However, larger sample sizes exceeding 100 are preferred to enhance the robustness of the findings.^21^

## RESULTS

We sent the survey to 1,434 medical doctors, and 425 fully completed it, resulting in a response rate of 29.6%. Among the respondents, 215 (50.6%) were female and 210 (49.4%) were male. Of the participants, 193 (45.4%) identified as emergency physicians, 156 (36.7%) as EM residents, and 76 (17.9%) as medical doctors without a specialty. Additionally, 38 participants held an academic title. The majority (94.5%) of respondents reported working more than 40 hours per week. Table 1 summarizes the descriptive sociodemographic statistics of the study population.

**Table 1.** Characteristics of the study group.

Sociodemographic variables	n (%)
Sex	
Male	215 (50.6)
Female	210 (49.4)
Profession	
Emergency physician	193 (45.4)
EM resident	156 (36.7)
Medical doctor	76 (17.9)
Title	
Faculty members	38 (9.0)
Academic staff	156 (36.6)
Non-academic staff	231 (54.4)
Work experience (years)	
<2	83 (19.5)
2–5	109 (25.6)
6–10	111 (26.1)
11–15	61 (14.4)
>15	61 (14.4)
Institution	
Private hospital	13 (3.1)
State hospital	122 (28.7)
City hospital	45 (10.6)
University hospital	85 (20.0)
Educational and research hospital	160 (37.5)
Weekly hours of work	
<40	23 (5.4)
40–72	330 (77.6)
>72	72 (16.9)

This table presents the distribution of sociodemographic variables among emergency physicians who participated in the study. The data includes information on sex, professional role, academic title, years of work experience, type of institution where they are employed, and their weekly working hours. Each category and subcategory is provided with the number of individuals and the corresponding percentage of the total participant pool.

*ED*, emergency department; *EM*, emergency medicine.

We used Trockel’s cut points to analyze the responses, which indicated that only 9.6% of respondents were considered professionally fulfilled. Conversely, a significant 79.1% of respondents reported experiencing burnout. The content validity assessment, which employed both I-CVI index values and the S-CVI/average relevance (Ave), yielded excellent content validity. All items demonstrated an I-CVI value exceeding 0.80, with the S-CVI/ Ave reaching 0.92. The mean scores (standard deviation) for the items ranged from 3.34 (2.94) to 6.47 (2.62), as shown in Table 2. The Cronbach α score for the professional fulfillment scale and the overall burnout scale were 0.87 and 0.90, respectively. The Cronbach α values for each dimension are presented in Table 3.

**Table 2.** Mean scores and standard deviations of the survey tool items.

Survey tool items	Mean (SD)
Professional fulfillment	4.27 (2.29)
1. I feel happy at work	3.53 (2.65)
2. I feel worthwhile at work	3.34 (2.94)
3. My work is satisfying to me	3.97 (2.84)
4. I feel in control when dealing with difficult problems at work	4.11 (2.90)
5. My work is meaningful to me	5.74 (3.10)
6. I’m contributing professionally in the ways I value most	4.94 (3.10)
Overall burnout	5.26 (2.18)
Burnout: professional exhaustion	5.98 (2.28)
1. A sense of dread when I think about the work I have to do	4.92 (2.90)
2. Physically exhausted at work	6.47 (2.62)
3. Lacking in enthusiasm at work	6.42 (2.92)
4. Emotionally exhausted at work	6.12 (3.06)
Burnout: interpersonal disengagement	4.78 (2.48)
1. Less empathetic with my patients	4.74 (2.89)
2. Less empathetic with my colleagues	3.92 (2.92)
3. Less sensitive to others’ feelings/emotions	5.04 (2.98)
4. Less interested in talking with my patients	5.43 (3.05)
5. Less connected with my patients	5.38 (3.04)
6. Less connected with my colleagues	4.20 (3.10)

This table presents the mean scores and SDs for items related to professional fulfillment and burnout as assessed by the Turkish Professional Fulfillment Index.

**Table 3.** Internal consistency of the dimension of the Turkish Professional Fulfillment Index.

	Cronbach α		
Scale	This study	Original	Japanese study
Professional fulfillment	0.87	0.91	0.91
Overall burnout	0.90	0.92	NA
Professional exhaustion	0.84	0.86	0.80
Interpersonal disengagement	0.91	0.92	0.90

The table displays the internal consistency of the dimensions of the Turkish Professional Fulfillment Index in this study, measured by the Cronbach α, in comparison to the original scale and results from the Japanese study. The results of the Brazilian study are not included in this table since only global Cronbach α of the PFI (0.95) was cited by Silva-Junior et al.^13^ NA: not applicable (The Japanese study did not provide the Cronbach’s α of overall burnout).

The CFA run on the initial model with three factors and 16 items (Model 1) demonstrated low CFI and TLI and did not meet criteria for goodness of fit. Factor loadings of all items were greater than 0.40; therefore, no item was removed from the initial model. All factor loadings for each item are presented in Table 4. Index modifications suggested by CFA were applied subsequently to improve the model fit. After index modifications, Model 2 was significantly improved with acceptable CFI, TLI and RMSEA (Table 5, Figure).

**Table 4.** Factor loadings for each item of the Turkish Professional Fulfillment Index.

		Standardized factor loadings	
Factors	Items	Model 1	Model 2
Professional fulfillment	1. I feel happy at work	0.89	0.90
	2. I feel worthwhile at work	0.82	0.83
	3. My work is satisfying to me	0.78	0.78
	4. I feel in control when dealing with difficult problems at work	0.63	0.63
	5. My work is meaningful to me	0.61	0.58
	6. I am contributing professionally in the ways I value most	0.62	0.59
Burnout: professional exhaustion	1. A sense of dread when I think about work, I have to do	0.48	0.45
	2. Physically exhausted at work	0.71	0.70
	3. Lacking in enthusiasm at work	0.92	0.93
	4. Emotionally exhausted at work	0.89	0.89
Burnout: interpersonal disengagement	1. Less empathetic with my patients	0.82	0.83
	2. Less empathetic with my colleagues	0.64	0.65
	3. Less sensitive to others’ feelings/emotions	0.86	0.86
	4. Less interested in talking with my patients	0.88	0.88
	5. Less connected with my patients	0.90	0.91
	6. Less connected with my colleagues	0.59	0.56

This table presents the standardized factor loadings for each item of the Turkish Professional Fulfillment Index, as measured in two different models (Model 1 and Model 2). Model 1: before modification index; and Model 2: after modification index.

**Table 5.** Confirmatory factor analysis: models’ goodness of fit of the Turkish Professional Fulfillment Index and other versions.

	# of items	χ^2^/df	*P*	CFI	TLI	RMSEA	AIC
Model 1	16	5.963	<0.001	0.890	0.869	0.108	672.301
Model 2	16	2.894	<0.001	0.960	0.950	0.067	358.761
Brazilian version	16	3.498	<0.001	0.950		0.08	
Japanese version	16		<0.001	0.897	0.909	0.085	

This table summarizes the results of the confirmatory factor analysis (CFA) conducted to assess the goodness of fit for various models of the Turkish Professional Fulfillment Index and its comparisons with other international versions. χ^2^/df: chi-square/degree of freedom.

*AIC*, Akaike information criterion; *CFI*, comparative fit index; Model 1, before modification index; Model 2, after modification index; *RMSEA*, root mean square error of approximation; *TLI*, Tucker-Lewis Index.

**Figure.**
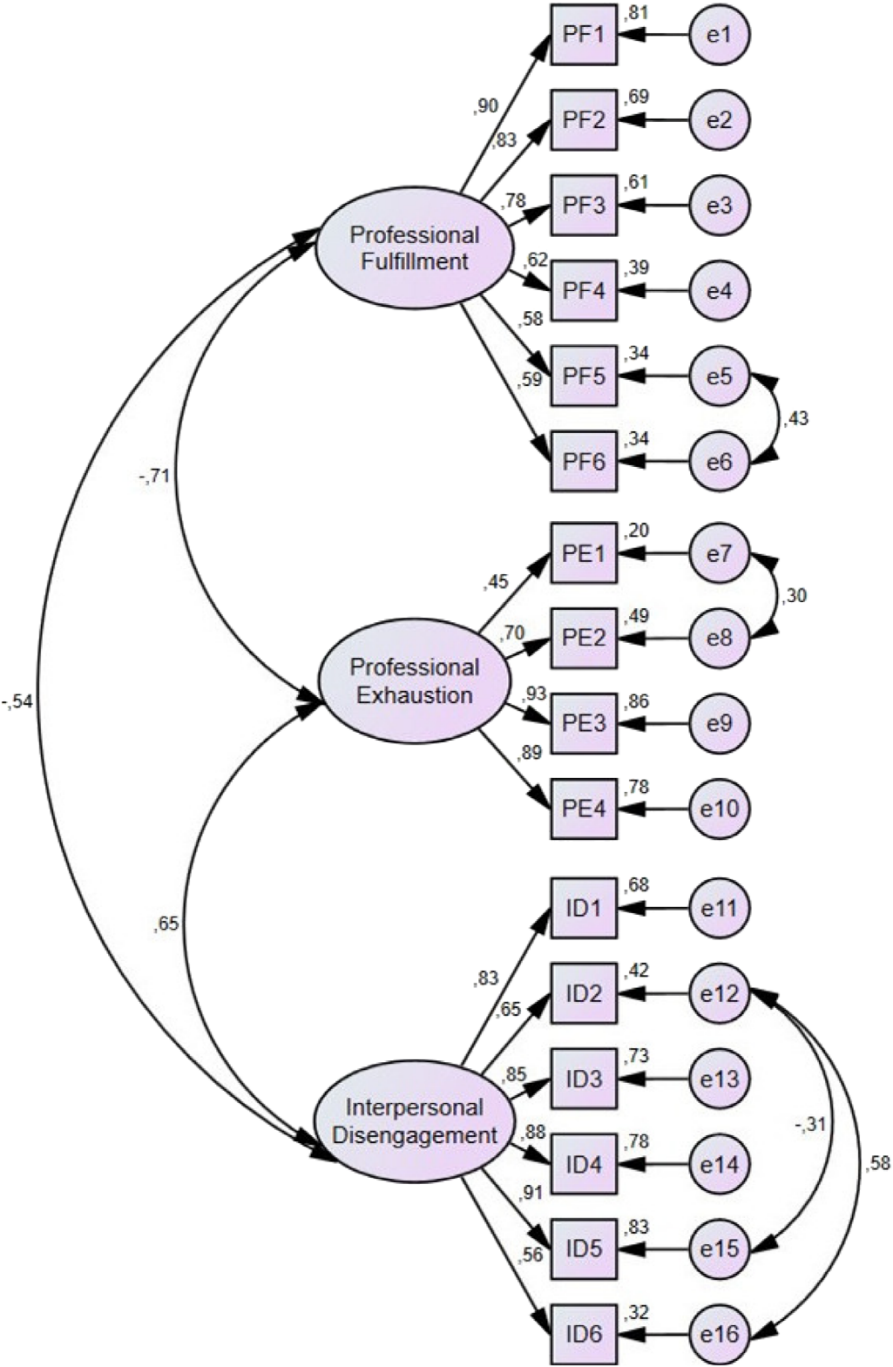
Confirmatory factor analysis of the Turkish Professional Fulfillment Index, Model 2 (after modification index). This figure presents the confirmatory factor analysis for the Turkish Professional Fulfillment Index. The model depicts the interrelationships between three latent constructs. The observed variables associated with the constructs of professional fulfillment (PF), professional exhaustion (PE), and interpersonal disengagement (ID) are presented.

## DISCUSSION

The T-PFI has demonstrated itself to be a robust and reliable measurement tool among emergency physicians in Turkey. Our study evaluated the T-PFI’s reliability and validity, revealing high internal consistency, strong factor loadings, and CFA results that align with international standards.^11^ This is significant given that previous cross-cultural adaptation studies of the PFI were conducted in Brazil and Japan, but not in Turkey.^12^ ^,^ ^13^ Our diverse participant group, consisting of physicians with various demographic characteristics and professional roles, enhances the generalizability of our findings. This diversity provides valuable insights into the experiences of emergency physicians, similar to studies conducted in other contexts. For instance, Asaoka et al included healthcare professionals from disaster medical assistance teams in Japan, while Silva-Junior et al focused on workplace physicians in Brazil.^12^ ^,^ ^13^

In our study, we achieved a response rate of 29.6%. Various factors, such as the high workload of emergency physicians, may have contributed to this response rate. According to Phillips et al, response rates in surveys of health professions trainees vary widely, ranging from 26.6–100%, with multi-institutional surveys typically having lower response rates compared to single-institution surveys.^22^ Our study, being multi-institutional, also faced this challenge. To improve the response rate, we employed multiple methods, including emails, social media groups, and internal communication platforms, such as online messaging systems, and we sent reminders to participants. By employing these strategies, we aimed to maximize participation and reduce non-response bias, ensuring that our findings accurately reflect the perspectives of a broad range of healthcare professionals within EM.

Based on our survey results, we found that emergency physicians in Turkey experience high levels of burnout. Only 9.6% of participants reported professional fulfillment, while 79.1% were likely experiencing burnout based on Trockel’s cut-off points. This finding is consistent with previous research that has linked the field of EM to high rates of burnout, attributed to the challenging nature of the specialty.^3^ ^–^ ^5^ ^,^ ^23^ ^–^ ^29^ For instance, a meta-analysis on burnout prevalence and risk factors among emergency healthcare workers found that Turkey has the highest prevalence of high emotional exhaustion.^25^

A cross-sectional survey study conducted among faculty members of the Academic Emergency Medicine Association, which also used the PFI scale, yielded intriguing results.^30^ In that study, 38.7% of participants reported feeling satisfied with their occupation, while 39.1% reported experiencing burnout. These results indicate a lower prevalence of burnout compared to our study. The discrepancy may be attributed to the fact that the sample included solely ED faculty members, whereas our study group consisted of a heterogeneous representation of emergency physicians, including residents and attending physicians. Consequently, our findings are more generalizable due to the diversity of participants, providing valuable insights into the experiences of clinicians in EDs.

The internal consistency of the T-PFI was robust, with the Cronbach α for professional fulfillment at 0.87 and for overall burnout at 0.90. These values are comparable to those reported in the original US validation study and the Japanese study.^10^ ^,^ ^12^ In the Brazilian validation study, only a global Cronbach α of 0.95 was reported, indicating high reliability across different cultural contexts.^13^

The initial CFA for the T-PFI indicated low CFI and TLI, which did not meet the criteria for goodness of fit. After applying index modifications, the fit indices significantly improved, with Model 2 achieving a CFI of 0.960 and an RMSEA of 0.067. These results align with those of the Japanese study, which also demonstrated acceptable fit indices after modifications.^12^ The Brazilian study similarly reported a CFI of 0.950 and RMSEA of 0.08, underscoring the model’s robustness across different populations.^13^ In the Japanese validation study, the CFA model fit was modest, and exploratory factor analysis confirmed a three-factor structure similar to the original scale. Our study’s factor loadings ranged from 0.45–0.93, confirming the T-PFI’s structural validity.

Although we did not assess convergent validity with external measures, the findings from the Japanese study of the PFI demonstrated significant positive correlations between professional fulfillment and quality of life, as well as between burnout subscales and depressive symptoms.^12^ This aligns with literature suggesting that higher levels of professional fulfillment are associated with better quality of life, while higher levels of burnout are linked to increased depressive symptoms.^10^

The use of the PFI in different healthcare settings, including pharmacists and physicians, has demonstrated its versatility and reliability.^31^ ^,^ ^32^ For instance, a study on US pharmacists found that professional fulfillment was associated with demographics and work settings, with community pharmacists reporting the lowest fulfillment and highest burnout.^32^ This pattern is similar to our findings, where emergency physicians exposed to high stress and long working hours reported significant burnout and low professional fulfillment.

The results of this study have significant implications for both clinical practice and healthcare policy. The high prevalence of burnout among emergency physicians in Turkey highlights the urgent need for systematic interventions. Healthcare administrators should integrate the T-PFI into regular evaluations to monitor physician well-being proactively. This could lead to early identification of burnout symptoms, allowing for timely interventions. For instance, Bodenheimer and Sinsky emphasize the importance of the quadruple aim, which includes improving clinicians’ work life as a crucial component of enhancing patient care.^9^ Additionally, Shanafelt et al found that addressing physician burnout can significantly improve patient care quality and reduce healthcare costs.^3^ ^,^ ^7^

The findings of this study can inform policy decisions at both organizational and governmental levels. Policies designed to reduce work hours, provide mental health resources, and create a supportive work environment are justified by the data indicating high burnout levels. Future research should investigate the longitudinal impact of burnout and professional fulfillment on career longevity and patient care quality among emergency physicians. Collaborative efforts across countries could further refine the T-PFI, making it a global standard for assessing physician well-being.

## LIMITATIONS

A notable limitation of this study is its focus on emergency physicians, which may limit the generalizability of the findings to other medical specialties or departments. Future research should include a broader range of healthcare professionals across various specialties to enhance the comprehensiveness of the findings. Additionally, we did not compare the demographic characteristics of respondents and non-respondents. Future studies might benefit from such comparisons to better address non-response bias. Longitudinal studies are also recommended to understand the temporal dynamics of professional fulfillment and burnout. Investigating organizational factors could provide deeper insights into creating healthier work environments for physicians.

## CONCLUSION

In this study we successfully translated, adapted, and validated the Turkish Professional Fulfillment Index, establishing its reliability as a tool for assessing professional fulfillment and burnout among medical doctors in Turkish EDs. The findings highlight a significant prevalence of burnout, emphasizing the need for targeted interventions to enhance physician well-being in Turkish healthcare settings. By providing a reliable measure of professional fulfillment and burnout, the T-PFI can guide healthcare organizations in developing programs to improve physicians’ job satisfaction and overall well-being.

## Supplementary Information




