# Supplementary material for: Validation of the Turkish Version of the Professional Fulfillment Index
Source: West J Emerg Med. 2024 Sep 25;25(6):958–65. doi: 10.5811/westjem.21199 (PMC11610730; doi:10.5811/westjem.21199)
Supplement: Supplementary file 1 [file wjem-25-958-s001.docx]

**Appendix A. Original and Turkish Version Codes and Titles for Professional Fulfillment Index Items**

| Code | Original | Turkish Version |
| --- | --- | --- |
| Title | Professional Fulfillment Index | Mesleki Tatmin Endeksi |
| Dimension 1 | Professional Fulfillment | Mesleki Tatmin |
| Opening 1 | How true do you feel the following statements are about you at work during the past two weeks? | Geçtiğimiz iki hafta boyunca iş yerinde sizinle ilgili aşağıdaki ifadelerin ne kadar doğru olduğunu düşünüyorsunuz? |
| Likert 1 | not at all true; somewhat true; moderately true; very true; completely true | Hiç doğru değil; biraz doğru, orta derecede doğru; çok doğru, tamamen doğru |
| PF 1 | I feel happy at work | İş yerinde kendimi mutlu hissediyorum. |
| PF 2 | I feel worthwhile at work | İş yerinde kendimi değerli hissediyorum. |
| PF 3 | My work is satisfying to me | İşim beni tatmin ediyor. |
| PF 4 | I feel in control when dealing with difficult problems at work | İşyerinde zor sorunlarla uğraşırken kontrolün elimde olduğunu hissediyorum. |
| PF 5 | My work is meaningful to me | İşim benim için anlamlı. |
| PF 6 | I’m contributing professionally (e.g. patient care, teaching, research, and leadership) in the ways I value most | Profesyonel olarak en çok değer verdiğim alanlarda (örneğin hasta bakımı, eğitim, araştırma ve liderlik) katkı sağlıyorum. |
| Dimension 2 | Burnout (work exhaustion and interpersonal disengagement scales) | Genel Tükenmişlik (İş bitkinliği ve Kişilerarası Kopukluk Skalaları) |
| Opening 2 | To what degree have you experienced the following? | Aşağıdakileri ne ölçüde deneyimlediniz? |
| Likert 2 | not at all; very little; moderately; a lot; extremely | Hiç değil; Çok az, Orta derecede; Çok, Son derece fazla |
| Opening PE | During the past two weeks I have felt… | Geçtiğimiz iki hafta boyunca... |
| PE 1 | A sense of dread when I think about work, I have to do | Yapmam gereken işleri düşündüğümde bir korku hissettim. |
| PE 2 | Physically exhausted at work | İş yerinde fiziksel olarak bitkin hissettim. |
| PE 3 | Lacking in enthusiasm at work | İş yerinde heves eksikliği hissettim. |
| PE 4 | Emotionally exhausted at work | İş yerinde duygusal olarak tükenmiş hissettim. |
| Opening ID | During the past two weeks my job has contributed to me feeling… | Geçtiğimiz iki hafta boyunca işim… |
| ID 1 | Less empathetic with my patients | Hastalarımla daha az empati kurmama neden oldu. |
| ID 2 | Less empathetic with my colleagues | Meslektaşlarımla daha az empati kurmama neden oldu. |
| ID 3 | Less sensitive to others’ feelings/emotions | Başkalarının hislerine/duygularına karşı daha az duyarlı olmama neden oldu. |
| ID 4 | Less interested in talking with my patients | Hastalarımla konuşmaya daha az ilgi duymama neden oldu. |
| ID 5 | Less connected with my patients | Hastalarıma daha az bağlı hissetmeme neden oldu. |
| ID 6 | Less connected with my colleagues | Meslektaşlarıma daha az bağlı hissetmeme neden oldu. |

**PF:** Professional Fulfillment, **PE:** Professional Exhaustion, ID**:** Interpersonal Disengagement
